# A novel method for demonstrating cold agglutinin disease: a case report

**DOI:** 10.1186/s13256-018-1573-7

**Published:** 2018-04-18

**Authors:** Thomas A. Vo, Zack Oakey, Yasir A. Khan, Donald S. Minckler

**Affiliations:** 1 Gavin Herbert Eye Institute, University of California, 850 Health Sciences Rd, Irvine, CA 92697 USA; 2 Division of Hematology and Oncology, University of California, Irvine, California 92697 USA

**Keywords:** Cold agglutinin disease, Anemia, Autoimmune, Conjunctival vessels

## Abstract

**Background:**

Cold agglutinin disease is a rare disorder characterized by an autoimmune hemolytic anemia occurring at low temperatures. Physical examination findings, often limited to acrocyanosis, are combined with a thermal amplitude test to help establish the diagnosis. Thermal amplitude testing determines the highest temperature at which the cold agglutination will occur and is an important parameter in diagnosing cold agglutinin disease.

**Case presentation:**

Here we describe a 57-year-old white man of German and Nicaraguan descent with known chronic cold agglutinin disease who presented to our ophthalmology clinic for evaluation of a cataract. During routine cataract surgery, the lowered temperature of the conjunctiva from intermittent flow of balanced salt solution at room temperature induced a cold agglutination reaction in conjunctival vessels easily visible under a surgical microscope.

**Conclusions:**

To the best of our knowledge, this method of demonstrating cold agglutinin disease has not been described in the literature and could easily be performed utilizing an ordinary slit lamp. This method could be used as an alternative and rapid screening method for cold agglutinin disease.

**Electronic supplementary material:**

The online version of this article (https://doi.org/10.1186/s13256-018-1573-7) contains supplementary material, which is available to authorized users.

## Background

Autoimmune hemolytic anemia cold agglutinin disease (CAD) is a rare disorder characterized by an immune reaction against red blood cell (RBC) self-antigens. “Cold agglutinin” describes the binding of the immunoglobulin (Ig) with erythrocytes at low temperatures, causing them to agglutinate and consequently induce hemolysis in those with CAD. CAD typically affects patient in their seventh decade of life, with clinical manifestations including livedo reticularis, Raynaud disease, and acrocyanosis [1–4]. Due to both its rarity and limited findings on physical examination, diagnosing CAD can be difficult.

Because many patients with cold agglutinins may never develop any symptoms, titer levels help determine which patients have clinically significant levels of cold agglutinin and which do not. Titer levels are not fully concordant though and may vary indirectly with disease severity, making it less reliable in some patients. Thermal activity, however, demonstrates the disorder and may be the most valuable test for preventing overdiagnosis of patients with CAD [1, 2]. Demonstrating thermal activity is important to determine the thermal amplitude, which is the highest temperature at which the cold agglutination reaction is observed [4]. Such testing not only confirms cold agglutinin activity, but also determines whether such activity is clinically significant [2].

Here we describe a 57-year-old white man with chronic CAD who presented to our ophthalmology clinic for evaluation of a cataract. During cataract surgery, it was observed that the lowered temperature environment produced by the intermittent flow of balanced salt solution (BSS) over his eye, which is done in routine surgery, induced a cold agglutination reaction in conjunctival vessels easily visible under a surgical microscope. To the best of our knowledge, this method of demonstrating CAD has not been described in the literature and could easily be performed utilizing an ordinary slit lamp as an alternative way to demonstrate thermal activity.

## Case presentation

Our patient is a 57-year-old white man of German and Nicaraguan descent with high myopia and previously diagnosed CAD. He works as a mechanical engineer with no significant past medical history, family history, or environmental history. His CAD was diagnosed 4 years prior, when he was experiencing cold-induced changes in his fingers, nose, feet, and with episodic dark urine when exposed to cold. A physical examination was significant for acrocyanosis and splenomegaly. His initial laboratory results included a total bilirubin of 5.3 mg/dL (reference ≤ 1.0 mg/dL), lactate dehydrogenase (LDH) 587 IU/L (reference ≤ 180 IU/L), creatinine 0.85 mg/dL (reference ≤ 1.30 mg/dL), alanine aminotransferase (ALT) 23 U/L (reference ≤ 63 U/L), aspartate aminotransferase (AST) 27 U/L (≤ 34 U/L), hemoglobin 11.0 g/dL (reference 14.0 to 18.0 g/dL), hematocrit (HCT) 39.5% (reference 42.0 to 52.0%), mean corpuscular volume (MCV) 93.8 fL (reference 80.0 to 94.0 fL), reticulocyte 5.2% (reference 0.4 to 2.5%), and cold hemagglutinins 40960. He was treated for 4 weeks with rituximab 375 mg/m^2^ weekly, to which he responded favorably. Since then, he has required a second course of rituximab for worsening of his disease, to which again he had a favorable response.

**Fig. 1:**
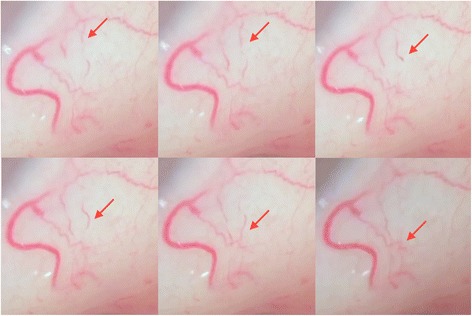
Interrupted blood flow in conjunctival vessels due to red blood cell agglutination (*red arrow*)


He presented to our ophthalmology clinic 7 months after his most recent rituximab treatment with symptoms of glare and decreased visual acuity and elected to undergo cataract surgery. At the time of presentation, he still had signs of acrocyanosis and splenomegaly and vital signs were within normal limits. During routine and uncomplicated surgery, it was noted that the temperature environment produced by intermittent flow of BSS over his eye induced a change in blood flow within the conjunctival vessels. Whereas under normal circumstances, RBCs are separate from their contiguous neighboring cells, our patient’s RBCs began to agglutinate, producing an unconventional finding of interrupted RBC collections or cassettes within the vasculature (see Fig. 1 and Additional file 1). The irregular clumping of RBCs, characteristic of CAD, was easily observable under a surgical microscope. We identified some degree of stasis in which the smaller vessels, particularly near the corneal limbus and anterior conjunctiva, did not show nearly as much flow as the posterior, larger vessels. The cataract surgery was otherwise uneventful and our patient had no complications during the intraoperative or postoperative period. At follow-up, he continued to have symptoms of CAD such as acrocyanosis and other temperature-induced skin changes. He is expected to undergo a third round of treatment with rituximab with the addition of high-dose prednisone for refractory CAD.

Additional file 1: Video demonstrating the cold agglutination reaction occurring in the conjunctival vessels (*red arrow*). The flow of blood is visibly interrupted. (MP4 49398 kb)


## Discussion and conclusions

Here we describe a 57-year-old white man with known history of CAD, who presented for cataract evaluation. During uncomplicated surgery, it was observed that the flow of BSS over the ocular surface induced the clumping of RBCs, characteristic of CAD. To the best of our knowledge, this finding has not been described or documented before.

CAD is a rare and poorly understood hematologic disorder. The term cold agglutinin arises from the finding that RBCs from blood samples of affected patients agglutinate without antiglobulin antisera in laboratory wells at 4 °C. Protein analysis has led to the discovery that the implicated IgM molecule is a 1 million Da protein, which binds RBCs at a higher affinity in low temperatures and is capable of spanning the distance between RBCs and overcoming the natural repulsive forces between cells within vessels [1]. The result is an irregular clumping of RBCs within the vessels, which is very distinct in appearance from physiological rouleaux formation.

Because of its rarity and limited physical examination findings, diagnosis is often late and difficult to make. Serum studies showing hyperbilirubinemia, elevated LDH, and positive Coombs testing for anti-C3 and negative anti-IgG are all suggestive, however, variably positive [1, 2]. Reviewing these diagnostic modalities, Chandesris *et al*. studied a group of 58 patients and found positivity for anti-C3 antibodies (74%), anti-C3d antibodies + IgG (22%), and IgG alone (3%) [3]. While these findings may suggest CAD, it is also important to evaluate titer levels and thermal activity to prevent over diagnosis. However, titer levels are less concordant and vary indirectly with disease severity. Stone *et al*. showed that only 14 of 172 (8%) patients with cold agglutinins displayed any clinical significance [5]. Thermal activity ultimately demonstrates disease activity [1, 2].

A practical screening procedure for CAD is to test the ability of the patient’s serum to agglutinate at 20 °C (or room temperature) [2]. If negative, CAD is unlikely, and if positive, further studies are needed to determine thermal amplitude. Thermal amplitude, which is the highest temperature at which the antibody will react with the antigen, appears to be the most important in regards to pathogenicity in CAD [4].

In the case described above, cold agglutination was observed by the intermittent flow of BSS over the eye during routine cataract surgery. While the differential includes other diagnoses such as sickle cell anemia, neoplasm, and cryoglobulinemia, given our patient’s known history of CAD, it is most likely that the changes in blood flow were attributable to CAD. This novel method of inducing cold agglutination in the conjunctival vessels could easily be performed utilizing an ordinary slit lamp. By testing the BSS at varying temperatures, determining thermal amplitude would be possible. Compared to the current methods of testing thermal activity, this method would have the advantage of being performed *in vivo*. This method could also be used as an alternative, inexpensive, and rapid screening method for CAD. This would circumvent unnecessary blood draws, laboratory testing, and the costs associated with them. A series of cases would help demonstrate the observations reported in this case of CAD. Future studies would also need to be conducted to test the sensitivity and specificity of using BSS in this manner though.

## Abbreviations

BSS: Balanced salt solution
CAD: Cold agglutinin disease
Ig: Immunoglobulin
LDH: Lactate dehydrogenase
RBC: Red blood cell
